# Impaired Amino Acid Metabolism and Its Correlation with Diabetic Kidney Disease Progression in Type 2 Diabetes Mellitus

**DOI:** 10.3390/nu14163345

**Published:** 2022-08-15

**Authors:** Huanhuan Zhu, Mengqiu Bai, Xishao Xie, Junni Wang, Chunhua Weng, Huifen Dai, Jianghua Chen, Fei Han, Weiqiang Lin

**Affiliations:** 1 Kidney Disease Center, The First Affiliated Hospital, Zhejiang University School of Medicine, Hangzhou 310003, China; 2 Key Laboratory of Kidney Disease Prevention and Control Technology, Institute of Nephrology, Zhejiang University, Hangzhou 310003, China; 3 The Fourth Affiliated Hospital, Zhejiang University School of Medicine, Jinhua 322000, China

**Keywords:** type 2 diabetes mellitus, diabetic kidney disease, metabolomics, amino acid metabolism, DNA methylation

## Abstract

Background: Metabolomics is useful in elucidating the progression of diabetes; however, the follow-up changes in metabolomics among health, diabetes mellitus, and diabetic kidney disease (DKD) have not been reported. This study was aimed to reveal metabolomic signatures in diabetes development and progression. Methods: In this cross-sectional study, we compared healthy (n = 30), type 2 diabetes mellitus (T2DM) (n = 30), and DKD (n = 30) subjects with the goal of identifying gradual altering metabolites. Then, a prospective study was performed in T2DM patients to evaluate these altered metabolites in the onset of DKD. Logistic regression was conducted to predict rapid eGFR decline in T2DM subjects using altered metabolites. The prospective association of metabolites with the risk of developing DKD was examined using logistic regression and restricted cubic spline regression models. Results: In this cross-sectional study, impaired amino acid metabolism was the main metabolic signature in the onset and development of diabetes, which was characterized by increased N-acetylaspartic acid, L-valine, isoleucine, asparagine, betaine, and L-methionine levels in both the T2DM and DKD groups. These candidate metabolites could distinguish the DKD group from the T2DM group. In the follow-up study, higher baseline levels of L-valine and isoleucine were significantly associated with an increased risk of rapid eGFR decline in T2DM patients. Of these, L-valine and isoleucine were independent risk factors for the development of DKD. Notably, nonlinear associations were also observed for higher baseline levels of L-valine and isoleucine, with an increased risk of DKD among patients with T2DM. Conclusion: Amino acid metabolism was disturbed in diabetes, and N-acetylaspartic acid, L-valine, isoleucine, asparagine, betaine, and L-methionine could be biomarkers for the onset and progression of diabetes. Furthermore, high levels of L-valine and isoleucine may be risk factors for DKD development.

## 1. Introduction

Diabetes mellitus (DM) is one of the fastest growing diseases, carrying persistent increases in the worldwide disease burden. Metabolic dysregulations have emerged as important signatures in the process of diabetes [1,2]. The application of high-throughput metabolomics has revealed a series of plasma metabolites prospectively associated with the biochemical process of diabetes. Studies have shown higher levels of branched-chain amino acids and aromatic amino acids in prediabetic and diabetic patients compared with normal subjects, and the underlying mechanism is that circulating amino acids may modulate insulin secretion and promote insulin resistance to promote pancreatic β-cell exhaustion [2,3]. Additionally, studies have shown that disorders of carbohydrate (fructose and glucose) metabolites or lipid (glycerophospholipids, sphingomyelins, and triglycerides) metabolites may prospectively correlate with diabetes risk [4,5].

Further, metabolomics has shown that dysregulated lipid and amino acid metabolism are associated with diabetic kidney disease (DKD) progression [6,7,8]. Recent studies revealed several candidate metabolites discriminating DKD from diabetes, showing that valine, xanthosine and 7-methyluric acid could be used to predict the development of renal injury in T2DM patients [9,10]. However, studies exploring the step-wise changes of metabolomics from healthy status to diabetes and then to DKD are limited, and researchers have commonly analyzed the metabolomic changes among DKD, DM and healthy controls at a single time point without following up DM patients to observe the onset of DKD. In this study, we compared untargeted metabolomic profiles between type 2 DM (T2DM) and healthy controls and between DKD and T2DM patients to identify altered metabolites with the same trend. Then, we followed up T2DM patients to observe whether they developed renal injury and analyzed the correlation between these altered metabolites with the onset of renal injury in order to find the potentially altered metabolites from healthy status to diabetes and then to DKD, as well as to evaluate the significance of these metabolites in predicting the development of DKD.

## 2. Materials and Methods

### 2.1. Human Subjects

**Figure 1:**
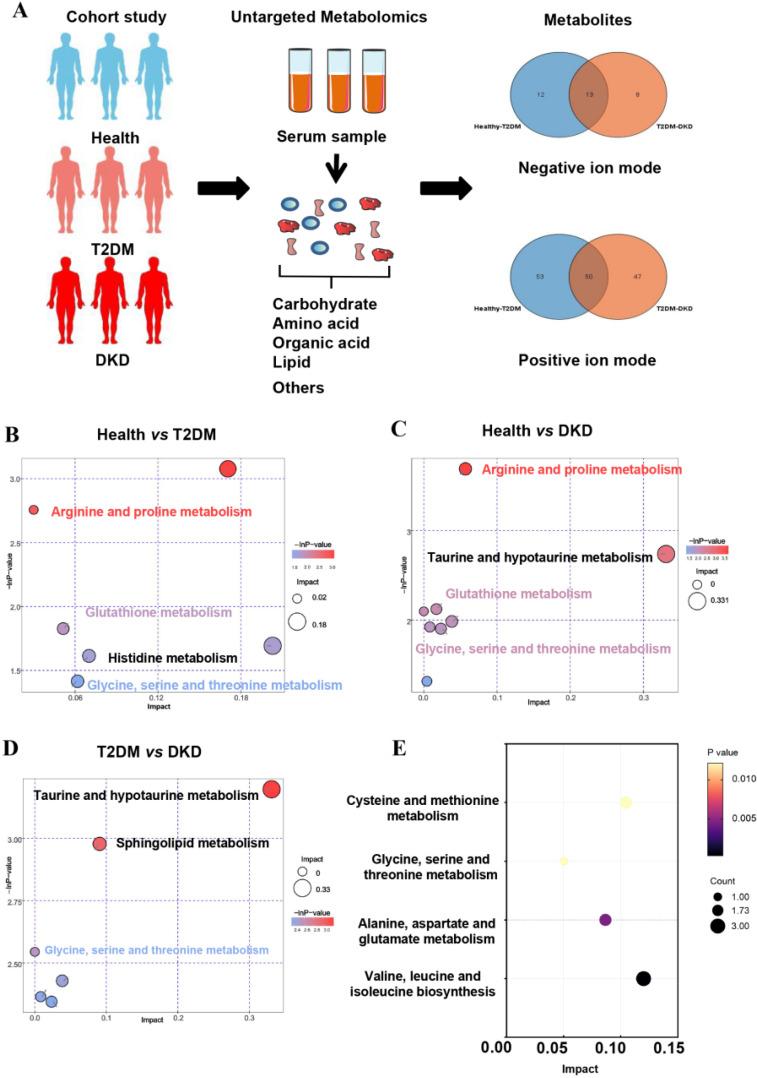
Overview of metabolic alterations in T2DM and DKD. (**A**) A Study design overview. Plasma metabolomics was collected for all study participants, and metabolic alterations were compared in both positive and negative ion modes. (**B**–**D**) Pathway enrichment analysis of significantly elevated metabolites in T2DM and DKD patients according to the KEGG pathway. (**E**) Differentially abundant metabolites in the onset and development of diabetes, stratified by KEGG pathways.

A total of 90 participants—30 T2DM patients (T2DM group), 30 DKD patients (DKD group), and 30 healthy volunteers as the control group (Health group)—were enrolled from the First Affiliated Hospital of Zhejiang University School of Medicine (Figure 1A). The T2DM patients were in line with the ADA criteria [11]. Their eGFR was ≥60 mL/min/1.73 m^2^, and their albuminuria (urine albumin–creatinine ratio, UACR) was ≤30 mg/g. The DKD patients met the American Diabetes Association (ADA) criteria, and their renal injury was proven by renal biopsy [11]. We excluded non-DKD patients; patients complicated with non-DKD such as IgA nephropathy, membranous nephropathy, interstitial kidney disease; patients with acute kidney injury; and patients with diseases that may affect albuminuria, such as urinary tract infection, urinary tumors, and cardiac insufficiency. Then, we followed up the patients in the T2DM group to observe whether they developed renal injury. The renal injury was defined as a rapid eGFR decline (an annual eGFR decline ≥3 mL/min/1.73 m^2^) or a diagnosis of DKD defined as eGFR declining to <60 mL/min/1.73 m^2^ or a persistently elevated urinary albumin level (UACR ≥30 mg/g). The eGFR slopes were calculated by the difference of eGFR values between baseline and the last follow-up time divided by the number of years. The current study was reviewed and approved by the institutional ethics committee of the First Affiliated Hospital of Zhejiang University, and all participants provided informed consent.

### 2.2. Measurement and Sample Collection

Data on age, gender, diabetic duration, and blood pressure were collected from medical records. The levels of fasting blood glucose, albumin, serum lipids, serum creatinine, and serum blood urea nitrogen were measured in a routine clinical laboratory. We used 24 h urine collection to assess the 24 h excretion of urinary albumin. The urinary albumin and creatinine levels were collected to calculate the albumin-to-creatinine ratio (ACR). eGFR was estimated using the Chronic Kidney Disease Epidemiology Collaboration (CKD-EPI) equation [12]. Patients with DKD were categorically divided by ACR into those with normal ACR (<30 mg/g Cr), microalbuminuria (30 to 300 mg/g Cr), and macroalbuminuria (>300 mg/g Cr).

Blood samples for metabolomics analysis were collected from each participant after overnight fasting, and then serum was isolated after centrifugation at 3000× *g* at 4 °C for 15 min. Serum samples were stored at −80 °C before further sample preparation and LC–MS analysis. Quality control (QC) samples were obtained by combining the serum sample from the different groups, and the measurements and analyses were repeated with the same LC–MS method.

### 2.3. Liquid Chromatography–Mass Spectrometry (LC–MS)

All serum samples were subjected to LC–MS metabolomics analysis on an ultra-high performance liquid chromatography (UHPLC) system (Thermo Fisher Scientific, Waltham, MA, USA) coupled with the TSQ Endura Triple Quadrupole Mass Spectrometer (Thermo Fisher Scientific, San Jose, CA, USA), according to previously described methods [13].

### 2.4. Metabolites Analysis

Raw data files were converted into the mzXML format using ProteoWizard software (version 3.0, Nashville, TN, USA) and processed by R package XCMS (version 3.2, La Jolla, CA, USA) for peak detection and normalization. The resulting three-dimensional data involving the peak number, sample name, and normalized peak area were fed to SIMCA software (version 14.1, MKS Data Analytics Solutions, Umea, Sweden) for principal component analysis (PCA) and orthogonal projections to latent structures-discriminant analysis (OPLS-DA). The metabolites were identified using the HMDB, PubChem, and ChEBI databases. The variable importance in projection (VIP) was used to identify differential metabolites in the DKD group relative to the T2DM group or the Health group. Metabolites with statistical significance (VIP > 1.0 and *p* < 0.05) were considered to be potential markers capable of differentiating DKD from T2DM or the control group. In addition, pathway enrichment analysis was conducted using the Kyoto Encyclopedia of Genes and Genomes (KEGG) database.

### 2.5. Statistical Analysis

All statistical analyses were performed using SPSS (version 25, Chicago, IL, USA), R (version 3.6.3, R Foundation for Statistical Computing, Vienna, Austria), and python (version 3.7, Python Software Foundation, Amsterdam, The Netherlands). Data were presented as the mean ± standard deviation for normal distribution and as median (interquartile range) for non-normal distribution. Data of normal distribution were compared using the independent sample *t*-test and the one-way analysis of variance, while the independent sample Kruskal–Wallis test was used for comparisons of non-normally distributed data. Pearson’s correlation was used to assess the association between metabolites and kidney function indicators. Moreover, receiver operating characteristic (ROC) analysis was performed to evaluate the diagnostic capability of identified potential metabolites. In the follow-up study, a logistic regression model was used to test associations of metabolites with rapid eGFR decline, and multivariate Cox analysis was performed to determine independent risk factors of diabetes prognosis. Restricted cubic spline (RCS) analysis was used to examine the nonlinear association of metabolites levels with DKD risk. Nonlinearity was tested using the likelihood ratio test. *p*-value < 0.05 was considered to be statistically significant.

## 3. Results

### 3.1. Metabolic Features in All Participants

The clinical characteristics of the Health, T2DM, and DKD groups were summarized in Table 1. There were no significant differences in age, sex, and duration of diabetes between the T2DM and DKD groups. Metabolomics showed positive and negative ion modes to detect all samples, and a total of 2433 serum metabolites were identified. To determine whether metabolites differed among the three groups, we performed principal components analysis (PCA) (Supplementary Figure S1). Moreover, an optimal orthogonal partial least squares-discriminant analysis (OPLS-DA) model was obtained using total area normalization to conduct the data analysis of metabolite profiling (Supplementary Figure S2). A permutation test was used to estimate the OPLS-DA model, while Q2Y and R2Y values close to 1 indicated that there was no overfitting. Supplementary Figure S3 indicates that the OPLS-DA model obtained high predictive features in the Health, T2DM, and DKD groups. According to the screening criteria (*p*-value < 0.05 and VIP values > 1), the statistical significance of metabolites was estimated to determine whether they were potential biomarkers between two groups in volcano plots (Supplementary Figure S4). Surprisingly, KEGG pathways showed the same paths of increased metabolite intensities among the progression of T2DM and DKD, with particular pathway focus on amino acid metabolisms, including arginine and proline metabolism, glutathione metabolism, and glycine, serine, and threonine metabolism (Figure 1B–D).

**Figure 2:**
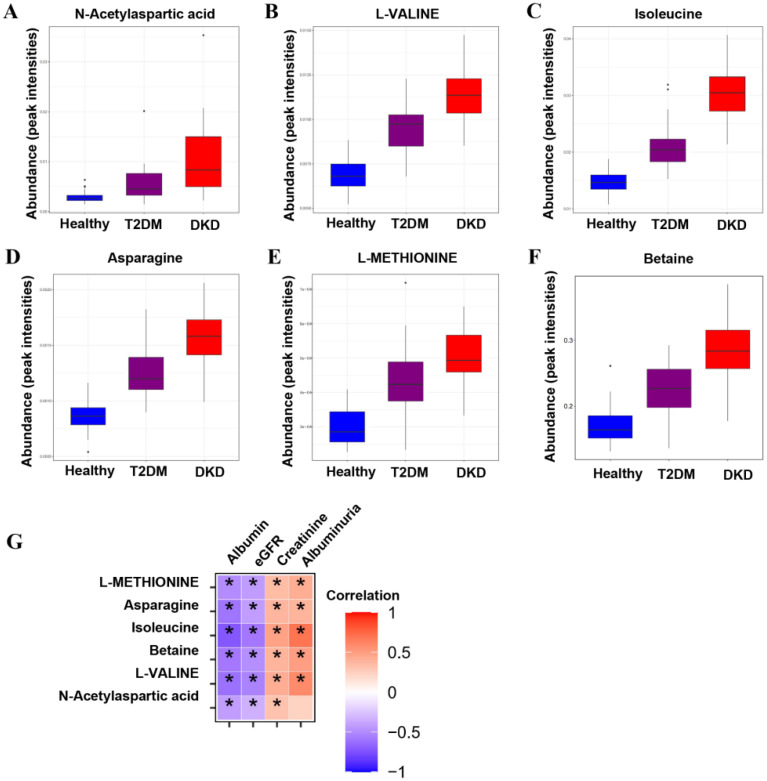
Metabolic differences across the Health–T2DM–DKD gradient. (**A**–**F**) Differential metabolites among Health, T2DM, and DKD groups, including the relative intensities of the six up-regulated overlapping metabolites from Health to T2DM and towards DKD. (**G**) The correlations between differential metabolites and clinical parameters. Significant *p*-value < 0.05. * *p* < 0.05.

To identify potential biomarkers in the progression of DM and DKD, we screened out metabolites that were elevated or gradually reduced from the Health group to the T2DM and DKD groups in the same direction with the PCA models (Supplementary Figure S1C,D). The metabolite-associated pathways were enriched among the progression of DM and DKD, where the majority were encoded in amino acid metabolism (Figure 1E), as described above. A total of 18 metabolites were identified (Supplementary Table S1), and six metabolites could be mapped into biochemical pathways. Compared with the Health group, the T2DM group had higher levels of N-acetylaspartic acid (NAA), L-valine, betaine, isoleucine, asparagine and L-methionine. The same trend was found between the T2DM and DKD groups (Figure 2A–F). Almost all metabolites were shown to be involved in amino acid metabolism, implying that amino acid metabolism may play an important role in the progression of DM and DKD. Moreover, the changing trend of other metabolites in the Health, T2DM, and DKD groups are shown in Supplementary Figure S5.

### 3.2. Correlation between Metabolites and Clinical Parameters

As shown in Figure 2G and Table 2, the metabolites of amino acids showed a broad range of correlations with clinical parameters. Serum albumin and eGFR were negatively correlated with levels of NAA, L-valine, betaine, isoleucine, asparagine and L-methionine. In contrast, serum creatinine and albuminuria were positively correlated with levels of L-valine, betaine, isoleucine, asparagine and L-methionine, and serum creatinine was also positively correlated with NAA. Based on the significant correlation of metabolites with renal function and proteinuria, we further analyzed the levels of metabolites in different subgroups of CKD stages and proteinuria. There were no significant differences in NAA, L-valine, betaine, isoleucine, asparagine, or L-methionine between the DKD patient groups with different CKD stages (Supplementary Figure S6). In different subgroups of degree of proteinuria, L-valine and betaine levels successively increased in the macroalbuminuria group compared with the normal albuminuria group (Supplementary Figure S7).

### 3.3. Validation of the Potential Biomarkers

**Figure 3:**
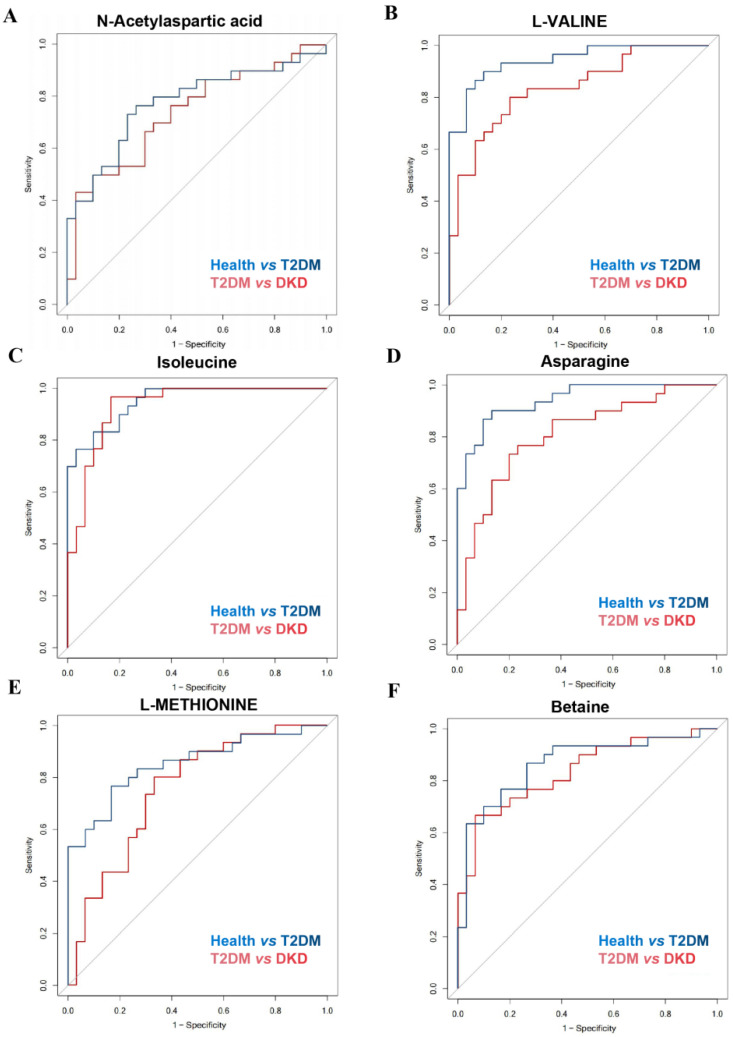
Discrimination ability of differential metabolites among Health, T2DM, and DKD groups. (**A**–**F**) ROC curves of differential metabolites for distinguishing health from T2DM patients and distinguishing T2DM patients from DKD patients.

To better understand the possible role of metabolites in distinguishing between the Health and T2DM groups or between the T2DM and DKD groups, ROC analysis was performed (Figure 3 and Table 3). Serum metabolite levels of NAA, L-valine, betaine, asparagine and L-methionine demonstrated accuracy and power in discriminating the DKD group from the T2DM group, as well as the T2DM group from the Health group, suggesting that metabolite differences may provide a way for identifying potential candidates for diabetes and DKD.

### 3.4. Correlation of Metabolites with Diabetes Progression

**Figure 4:**
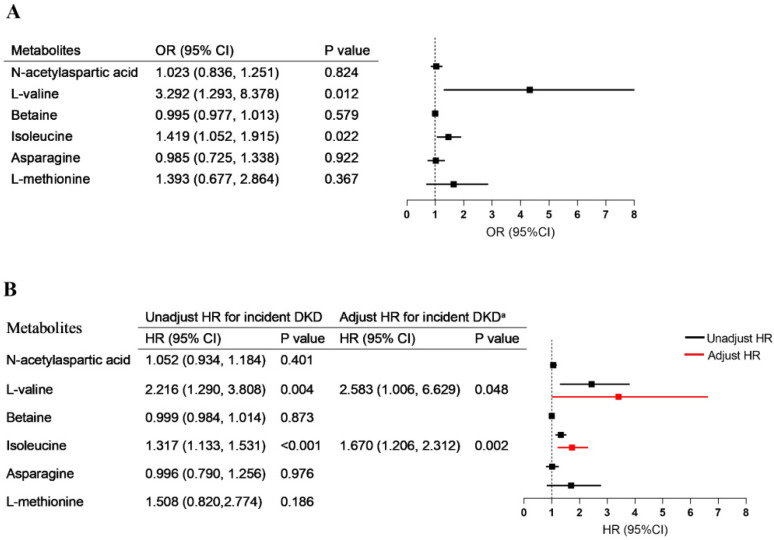
Association of metabolites with diabetes progression. (**A**) The associations of metabolic alterations (per one-point increment) with rapid eGFR decline. (**B**) The association between metabolic alterations (per one-point increment) and risk of new-onset diabetic kidney disease. Adjusted ^a^, without stratification, for age, sex, blood pressure, duration of diabetes, baseline eGFR, and albuminuria.

For patients in the T2DM group, after a median follow up of 69.00 (46.00, 71.00) months, 14 T2DM patients showed an annual eGFR decline ≥3 mL/min/1.73 m^2^ and 12 T2DM patients met the diagnosis criterion of DKD. In the logistic regression models, the concentrations of both L-valine and isoleucine were significantly associated with rapid eGFR decline (respectively, OR = 3.292, 95% CI = 1.293–8.378; OR = 1.419, 95% CI = 1.052–1.915) (Figure 4A). Univariable Cox regression analysis showed that high levels of L-valine and isoleucine were risk factors for DKD. After adjustment for the parameters of baseline age, sex, blood pressure, diabetes duration, eGFR, and albuminuria, the upregulated L-valine (HR = 2.583, 95% CI = 1.006–6.629, *p* = 0.048) and isoleucine (HR = 1.670, 95% CI = 1.206–2.312, *p* = 0.002) remained independent risk factors for the development of DKD in multivariate Cox regression (Figure 4B).

**Figure 5:**
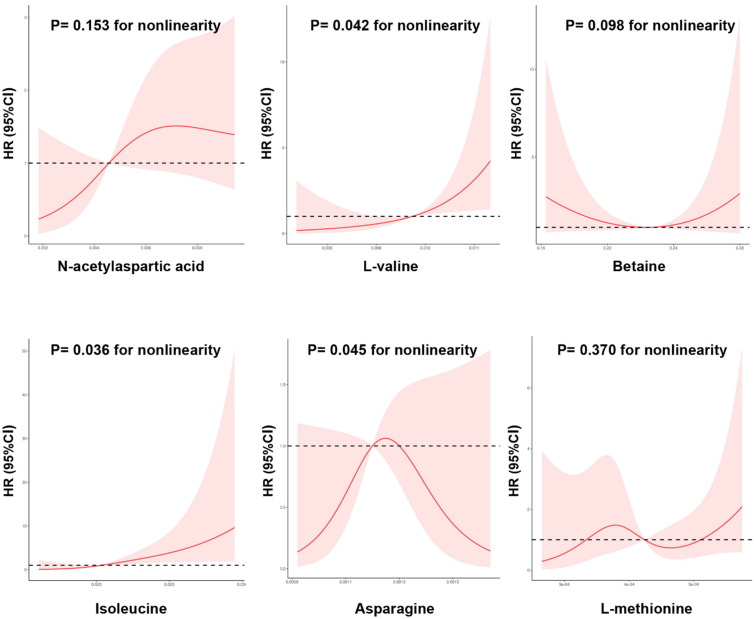
The nonlinear dose-dependent relationship between metabolic alterations and risk of new-onset diabetic kidney disease. Restricted cubic spline curve was carried out with 4 knots of baseline metabolic index. The solid line represents the association of metabolic index with DKD risk, and the shaded portion represents 95% CI estimation.

Moreover, restricted cubic spline analysis indicated a significant dose–response relationship between the risk of DKD and metabolites (Figure 5). A nonlinear association was observed between new-onset DKD and L-valine, isoleucine, and asparagine levels (*p* for nonlinearity < 0.05). That is, when levels of L-valine and isoleucine were relatively low, there was a negative correlation between L-valine and isoleucine levels and DKD risk; however, when L-valine and isoleucine exceeded certain thresholds (L-valine > 0.0097 peak intensities and isoleucine > 0.0205 peak intensities), the risks of new-onset DKD increased.

## 4. Discussion

Through untargeted metabolomics profiling, the present study focused on the continuously changing metabolites from healthy status to diabetes and then to DKD. Unique to the present research, we performed a prospective study to search for circulating metabolites associated with progressive eGFR decline and progression to DKD among T2DM patients. In this cross-sectional study, we found that upregulated amino acid metabolites levels were the main metabolic signatures in the occurrence and development of diabetes, which was characterized by increased NAA, L-valine, isoleucine, asparagine, and L-methionine levels. We also provided evidence that these candidate metabolites could distinguish the Health group and the DKD group from the T2DM group. Given this background, metabolites may offer insights into diabetes progression, so we evaluated associations of these previously identified metabolites with kidney function decline in a follow-up study. A key finding was that L-valine and isoleucine showed a strong independent effect on progressive renal decline in T2DM patients. Furthermore, the fact that higher baseline levels of L-valine and isoleucine were associated with an increased risk of DKD provides strong evidence that these metabolites are causally involved in diabetes development and progression. Next, we will discuss the biology of these metabolites and possible mechanisms through which they may contribute to the occurrence of diabetes and the progression of renal injury.

Amino acids are building blocks for all life forms, for which absorption and transportation are found in the small intestine, colon, liver, kidneys, and other tissues, therefore allowing amino acids to affect the growth and health of humans [14,15]. Branched chain amino acids (BCAAs)—referring to valine, leucine, and isoleucine—serve as important signaling molecules regulating the metabolism of proteins, glucose, and lipids, which play critical roles in energy homeostasis. Alterations in BCAA catabolism were found in diabetes decades ago, and BCAA metabolism is altered before the development of diabetes and is associated with the onset of it [16,17,18]. These high circulating levels of BCAAs are associated with an increased risk of type 2 diabetes, which has been verified in multiple cohorts [19,20,21]. Several mechanisms have been implicated in raising BCAA levels in insulin resistance (Supplementary Figure S8A). In the current study, we found that valine and isoleucine were gradually upregulated in T2DM and DKD patients. Consistent with our expectations, the different circulating concentrations of valine and isoleucine could distinguish healthy subjects and DKD patients from T2DM patients. These results are in agreement with the findings of previous studies, which confirmed the role of altered BCAA metabolism in the pathophysiology of diabetes [22,23]. A possible reason for these findings is that a defective BCAA catabolism regulates rapamycin (mTOR) pathway activation and insulin receptor substrate protein phosphorylation, which lead to insulin resistance and the accumulation of cytotoxic metabolites. Another noteworthy observation is that we found that high circulating levels of valine and isoleucine were correlated with decreased eGFR levels and increased albuminuria levels. Given these results and prior findings showing associations of amino acids with vascular complications in diabetes, we further explored whether amino acid levels were associated with adverse renal outcomes in T2DM patients. In a follow-up study of individuals with type 2 diabetes, we found that higher levels of both valine and isoleucine were associated with an increased risk of incident DKD. These findings supported the idea that BCAA metabolism potentially participates in the onset of diabetic kidney disease, and the pathophysiology underlying these associations is worthy of further investigation.

In addition to BCAAs, we also identified four other amino-acid-correlated metabolites that were significantly associated with diabetes and diabetic kidney diseases. The results of this study demonstrated the step-wise upregulation of circulating asparagine, NAA, L-methionine, and betaine in T2DM and DKD patients, which was significantly associated with renal function, serum albumin, and albuminuria. Our results also showed that these increased amino-acid-related metabolites could distinguish healthy subjects and DKD patients from T2DM patients, so they could be potential biomarkers for diabetes and DKD. Asparagine and aspartate can be converted to each other with metabolism fluctuation, and the roles of asparagine and aspartate homeostasis regulation have been highlighted in metabolic disorders. Previous research showed that an increasing asparagine-to-aspartate ratio was a risk for the incident of diabetes [24], which is consistent with the results obtained in our study. These findings could be explained by the idea that high levels of asparagine upregulate the mTORC1 pathway [25], which contributes to the development of insulin resistance [26]. However, other studies have shown that asparagine has an inverse association with diabetes risk [27], which was contrary to the results of our study. To date, the association of circulating asparagine levels and diabetes is still controversial in clinical settings. The alteration of asparagine metabolism has not been demonstrated in the pathogenesis of diabetes, so further studies investigating the underlying changes of asparagine in diabetes patients are still warranted. Moreover, NAA is involved in neuronal metabolism and downregulated in the brain of diabetes patients, which have been reported to be the key metabolites in the cognitive dysfunction of diabetes [28,29]. A previous study showed that lower levels of NAA in the brains of patients with diabetes indicate partial neuronal loss [30]. Despite these findings, circulating NAA levels in T2DM and DKD patients have received little attention, and our study demonstrated increased NAA levels in the blood of these patients. Although our findings have broadened insights into NAA metabolism in the progression of diabetes, the homeostasis of NAA in the circulation and cerebrum is worthy of further exploration.

DNA methylation could be involved in glucose metabolism, insulin resistance, and other conditions, leading to the pathogenesis of diabetes that continues to be an area of active research [31,32]. Several studies have shown that alterations of the DNA methylation in human tissues are of importance for the epigenome and may thereby affect gene expression and the pathogenesis of diabetes mellitus (Supplementary Figure S8B). Indeed, DNA methylation changes in diabetes may eventually contribute to vascular complications, including diabetic kidney disease. Methionine and betaine are vital methyl donors in DNA methylation, serving as cofactors for affecting methylation. Methionine is converted into S-adenosyl-l-methionine (SAM) and S-adenosyl-homocysteine (SAH), and the SAM/SAH ratio is known as a methylation regulation index. In line with the results of our study, the upregulation of circulating methionine and its catabolites has been observed in diabetes patients, and circulating methionine abundance could predict the risk of developing diabetes [33]. Methionine restriction attenuates glucose homeostasis, insulin sensitivity, oxidative stress, inflammation in diabetes, and this evidence highlights the idea that methionine is a potential contributor to the pathogenesis of diabetes [34]. Furthermore, methionine restriction has also been proven to activate renoprotective genes and improve kidney function decline associated with metabolic dysfunction [35,36], which is consistent with our findings. In addition, betaine is also involved in diabetes metabolic alterations through the control of the SAM/SAH ratio of DNA methylation. Betaine is upregulated in the blood of diabetes models and patients, so it is also considered a poor predictor for incident diabetes [37,38]. Nevertheless, a high circulating betaine concentration could contribute to diabetes complications, including diabetic kidney disease [39,40]. Therefore, more systematic studies are warranted to identify whether betaine is a potential target in diabetes conditions.

There were few limitations in the current study. First, a single untargeted metabolomics platform was used with relatively small-scale samples, and integration between metabolomics and proteomics should be utilized for systems biology information in the future. Second, despite the strong design of the study with a cross-sectional and follow-up cohort, an independent validation cohort is needed to confirm the presented findings because the number of current samples was insufficient to support correction for multiple comparisons. Finally, the molecular mechanisms of the studied metabolites are still uncertain, and mechanistic studies in diabetic models will be indispensable to understanding the roles of metabolites in future works.

## 5. Conclusions

We performed a cross-sectional study to identify consistently altered metabolites in diabetes and diabetic kidney diseases, and metabolomic changes in T2DM and DKD subjects were characterized by upregulated L-valine, isoleucine, asparagine, NAA, L-methionine, and betaine levels. The findings in a follow-up cohort suggested that L-valine and isoleucine were associated with an increased risk of incident DKD. This study highlights the idea that BCAA metabolism is disturbed in diabetes, which could be considered a biomarker for the prediction of DKD.

## Figures and Tables

**Table 1:** Clinical and biochemical parameters in Health, T2DM, and DKD groups.

	Health (n = 30)	T2DM (n = 30)	DKD (n = 30)	*p* ^a^	*p* ^b^
Age, years	39.20 ± 10.68	53.30 ± 17.00	50.70 ± 10.36	<0.001	0.478
Male sex	18 (60.00)	15 (50.00)	11 (36.70)	0.436	0.297
Duration of diabetes, years	/	6.24 ± 5.70	7.08 ± 4.69	/	0.535
Systolic blood pressure, mmHg	119.40 ± 11.62	119.47 ± 21.38	142.87 ± 21.60	0.988	<0.001
Diastolic blood pressure, mmHg	76.50 ± 6.12	75.53 ± 9.07	87.53 ± 11.82	0.630	<0.001
Total cholesterol, mmol/L	4.32 (3.73, 4.96)	3.83 (3.40, 4.55)	5.06 (3.26, 6.41)	0.017	0.012
Triacylglycerol, mmol/L	1.10 (0.81, 1.62)	1.03 (0.72, 2.04)	1.52 (1.06, 2.23)	0.784	0.025
HDL-cholesterol, mmol/L	1.13 ± 0.36	0.93 (0.82, 1.31)	1.24 ± 0.35	0.693	0.098
LDL-cholesterol, mmol/L	2.43 ± 0.60	1.95 (1.66, 2.68)	2.90 ± 1.35	0.145	0.012
Fasting glucose, mmol/L	4.70 ± 0.37	8.29 ± 3.35	6.77 ± 3.15	<0.001	0.081
HbA_1c_, %	/	10.81 ± 2.39	7.67 ± 2.35	/	<0.001
Creatinine, µmol/L	66.00 (59.00, 80.25)	59.00 (53.50, 83.00)	107.00 (84.50, 146.75)	0.325	<0.001
eGFR, ml/min/1.73 m^2^	104.01 ± 13.22	98.12 ± 21.77	63.23 ± 24.84	0.211	<0.001
Urea nitrogen, mmol/L	4.72 (4.15, 6.20)	5.20 (4.30, 6.40)	7.25 (6.23, 10.08)	0.520	<0.001
Uric acid, µmol/L	327.83 ± 119.46	280.07 ± 82.59	390.23 ± 113.53	0.077	<0.001
Albumin, g/L	47.95 (44.73, 50.15)	42.10 (40.00, 44.60)	30.75 (24.13, 40.03)	<0.001	<0.001
24 h urine protein, g	/	0.06 (0.04, 0.09)	3.22 (1.11, 5.14)	/	<0.001

^a^ *p*-value for comparing Health group with T2DM group. ^b^ *p*-value for comparing T2DM group with DKD group.

**Table 2:** Correlation analysis between clinical parameters and metabolites.

	eGFR		Serum Creatinine		Albuminuria		Serum Albumin	
Metabolites	Coefficient	*p*-Value	Coefficient	*p*-Value	Coefficient	*p*-Value	Coefficient	*p*-Value
N-acetylaspartic acid	−0.339	0.001	0.316	0.002	0.235	0.104	−0.423	<0.001
L-valine	−0.537	<0.001	0.419	<0.001	0.593	<0.001	−0.617	<0.001
Betaine	−0.488	<0.001	0.391	<0.001	0.498	<0.001	−0.585	<0.001
Isoleucine	−0.584	<0.001	0.482	<0.001	0.698	<0.001	−0.727	<0.001
Asparagine	−0.423	<0.001	0.383	<0.001	0.389	0.006	−0.599	<0.001
L-methionine	−0.427	<0.001	0.348	0.001	0.422	0.003	−0.497	<0.001

**Table 3:** The diagnostic power of different metabolite biomarkers in differentiating T2DM from Health controls or DKD from T2DM.

Metabolites	Pathway and Sub-Pathway	AUC (95% CI)	
		Health vs. T2DM	T2DM vs. DKD
N-acetylaspartic acid	Alanine, aspartate and glutamate metabolism	0.777 (0.655, 0.898)	0.739 (0.612, 0.866)
L-valine	Valine, leucine and isoleucine degradation	0.943 (0.889, 0.997)	0.834 (0.733, 0.936)
Betaine	Glycine, serine and threonine metabolism	0.863 (0.766, 0.960)	0.834 (0.732, 0.937)
Isoleucine	Valine, leucine and isoleucine degradation	0.951 (0.905, 0.997)	0.932 (0.869, 0.995)
Asparagine	Alanine, aspartate and glutamate metabolism	0.942 (0.889, 0.995)	0.809 (0.698, 0.920)
L-methionine	Cysteine and methionine metabolism	0.852 (0.754, 0.950)	0.753 (0.628, 0.878)

## Data Availability

The data underlying this study are available from the corresponding author upon reasonable request.

## Supplementary Materials

The following supporting information can be downloaded at: https://www.mdpi.com/article/10.3390/nu14163345/s1, Figure S1: PCA score plot in positive and negative ion mode among Health, T2DM, and DKD groups; Figure S2: OPLS-DA of serum metabolome; Figure S3: Cross-validation plot in OPLS-DA model; Figure S4: Volcano plots of candidate metabolites; Figure S5: Box plots of differential metabolites in serum samples; Figure S6: Detection of differentially expressed metabolites based on different CKD stages; Figure S7: Detection of differentially expressed metabolites based on different degree of proteinuria; Figure S8: The disturbance of amino acid metabolism pathway in the progression of diabetes; Table S1: The metabolites in Health, T2DM, and DKD subjects.
