# Supplementary material for: Impaired Amino Acid Metabolism and Its Correlation with Diabetic Kidney Disease Progression in Type 2 Diabetes Mellitus
Source: Nutrients. 2022 Aug 15;14(16):3345. doi: 10.3390/nu14163345 (PMC9415588; doi:10.3390/nu14163345)
Supplement: Supplementary file 1 [file nutrients-14-03345-s001.zip › nutrients-1858206-supplementary.pdf]

nutrients

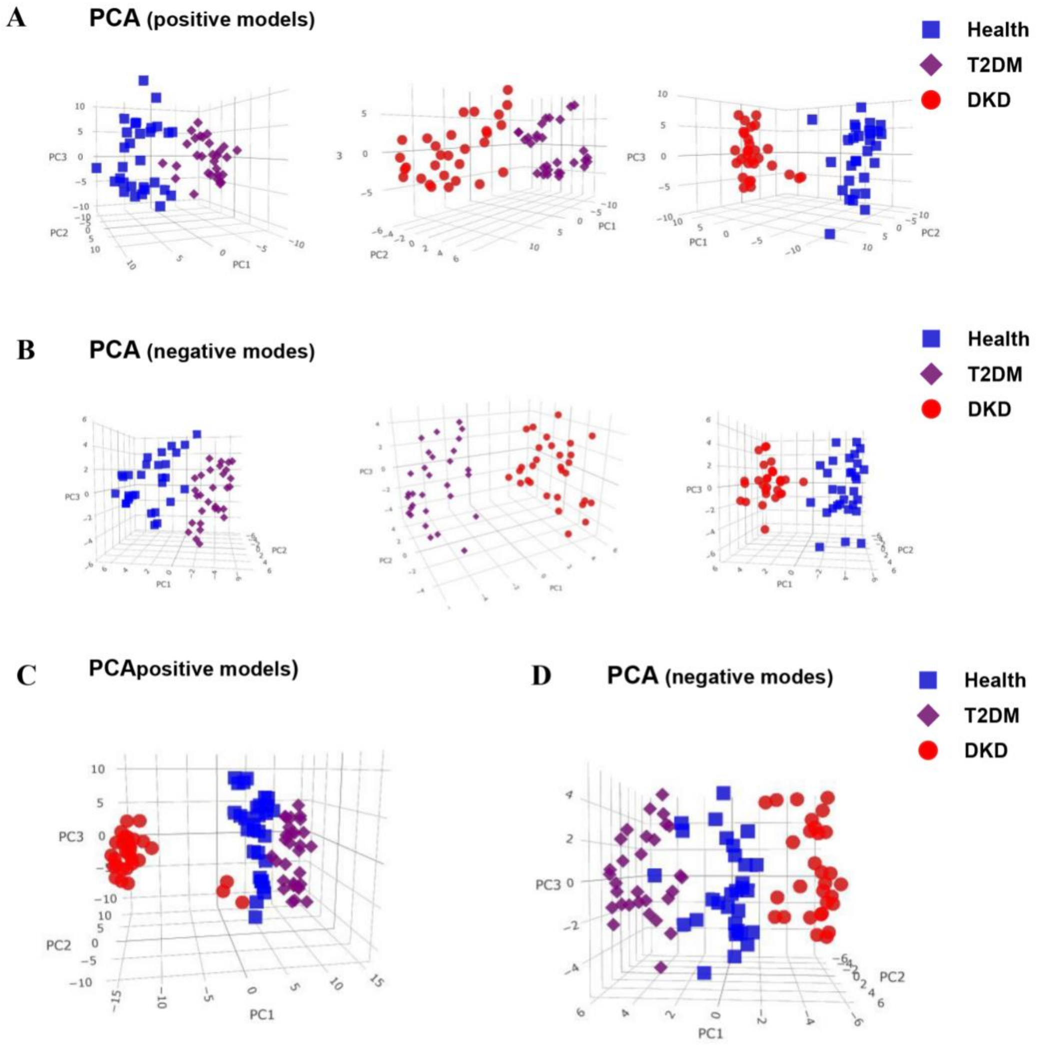

Figure S1. PCA score plot in positive and negative ion mode among Health, T2DM, and DKD groups.

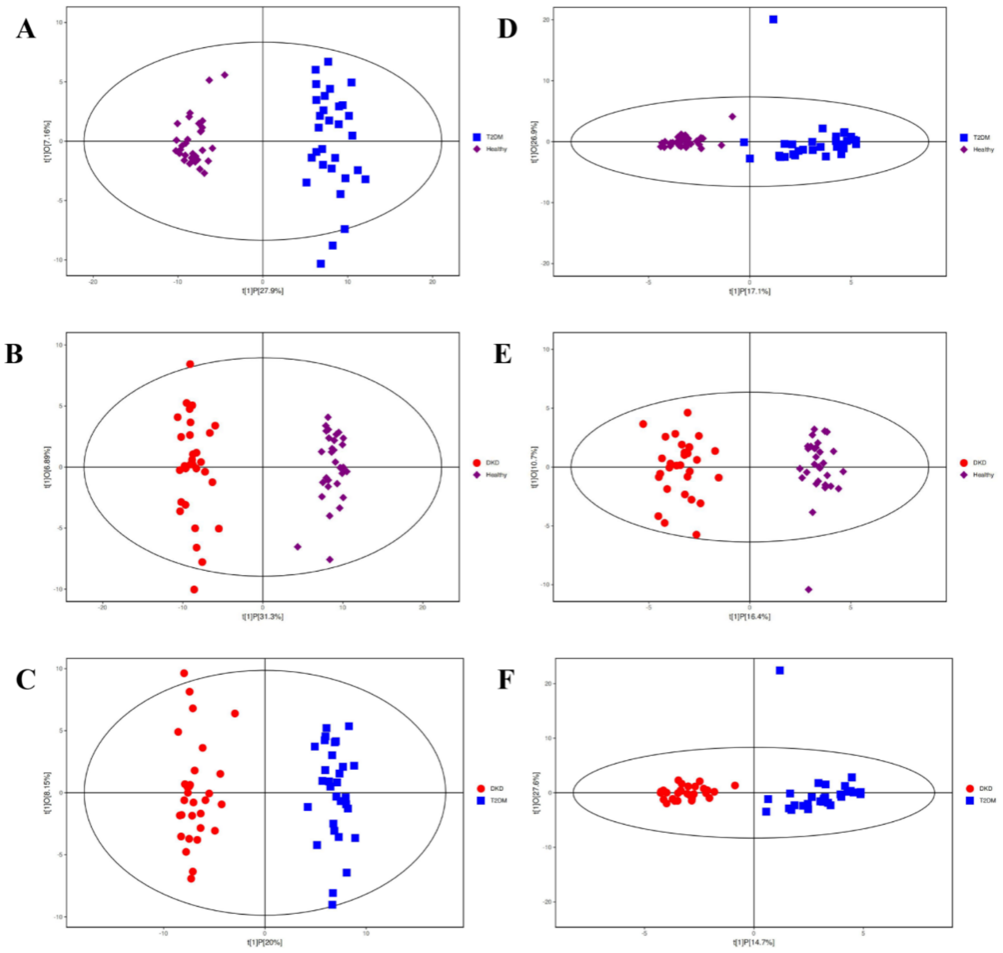

Figure S2. OPLS-DA of serum metabolome. (A-C) OPLS-DA score plot in positive ion mode. (D-F) OPLS-DA score plot in negative ion mode.

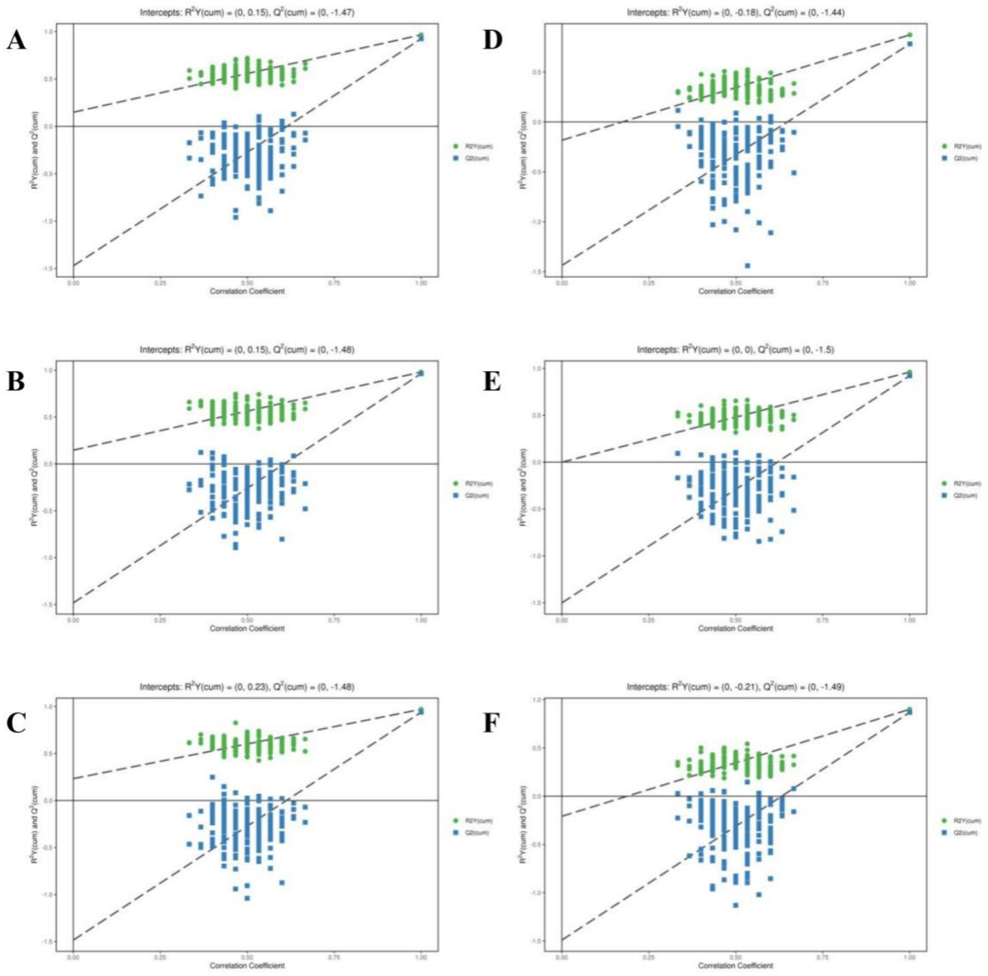

Figure S3. Cross-validation plot in OPLS-DA model. (A-C) OPLS-DA permutation test plot in positive ion mode. (D-F) OPLS-DA permutation test plot in negative ion mode.

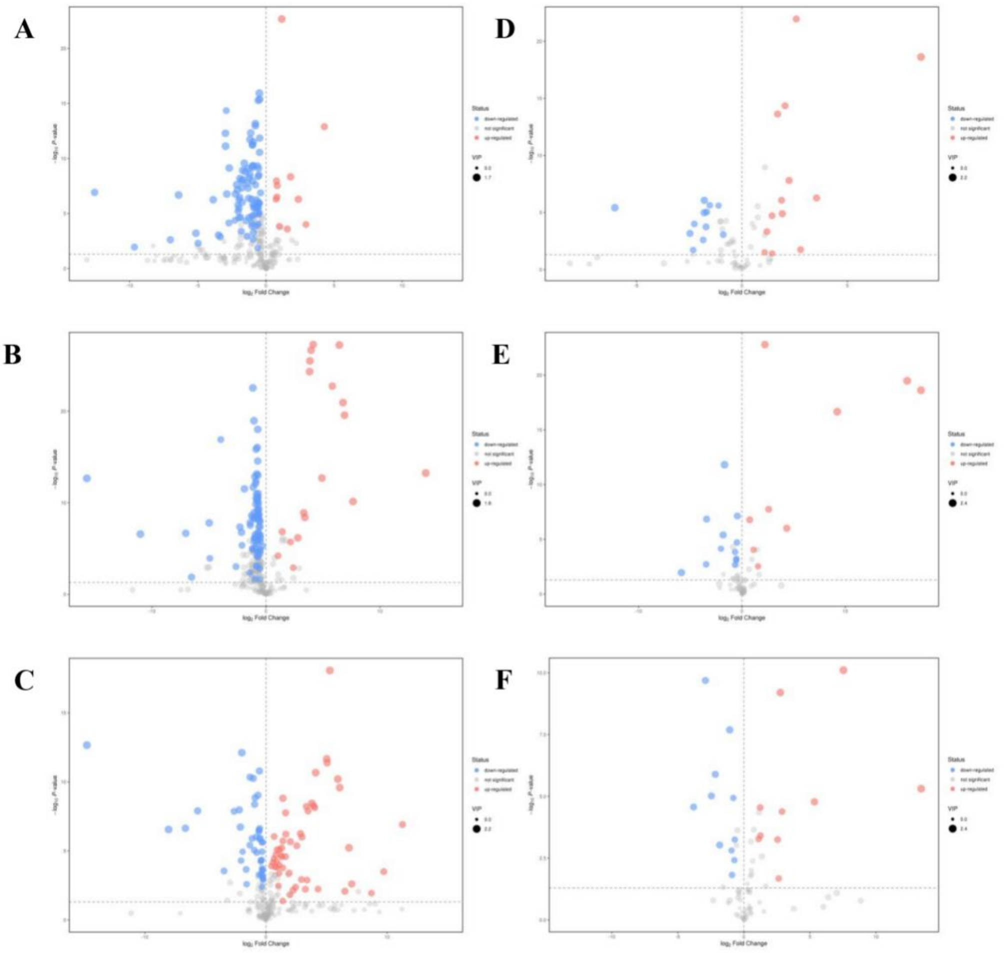

Figure S4. Volcano plots of candidate metabolites.(A-C) Volcano plots showing the results of pairwise comparisons of metabolites in positive ion mode (Health *vs* T2DM, Health *vs* DKD, T2DM *vs* DKD, respectively). (D-F) Volcano plots showing the results of pairwise comparisons of metabolites in negative ion mode (Health *vs* T2DM, Health *vs* DKD, T2DM *vs* DKD, respectively).

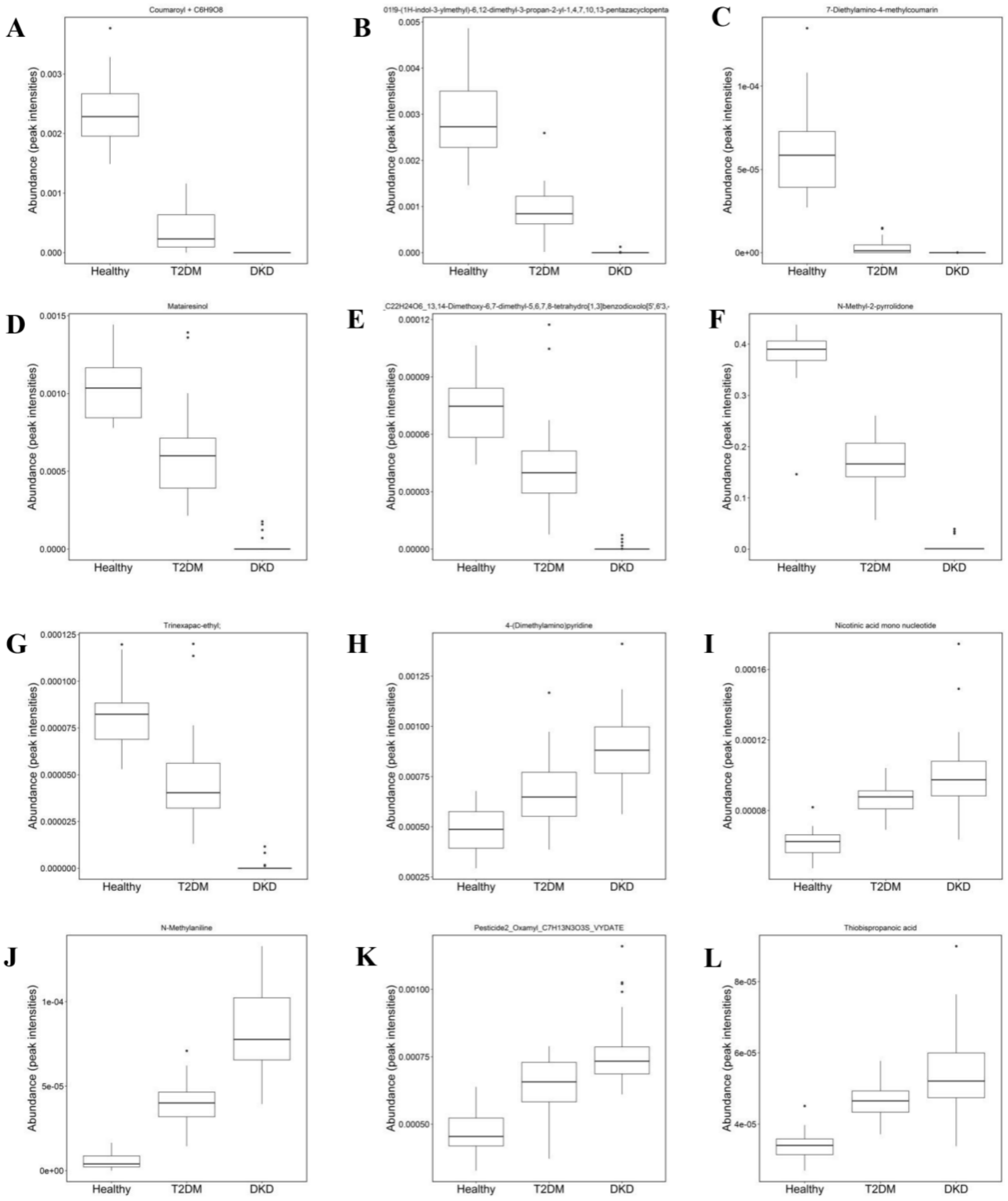

Figure S5. Box plots of differential metabolites in serum samples. (A-L) The relative intensities of other twelve up-regulated overlapping metabolites from Health to T2DM, and towards DKD.

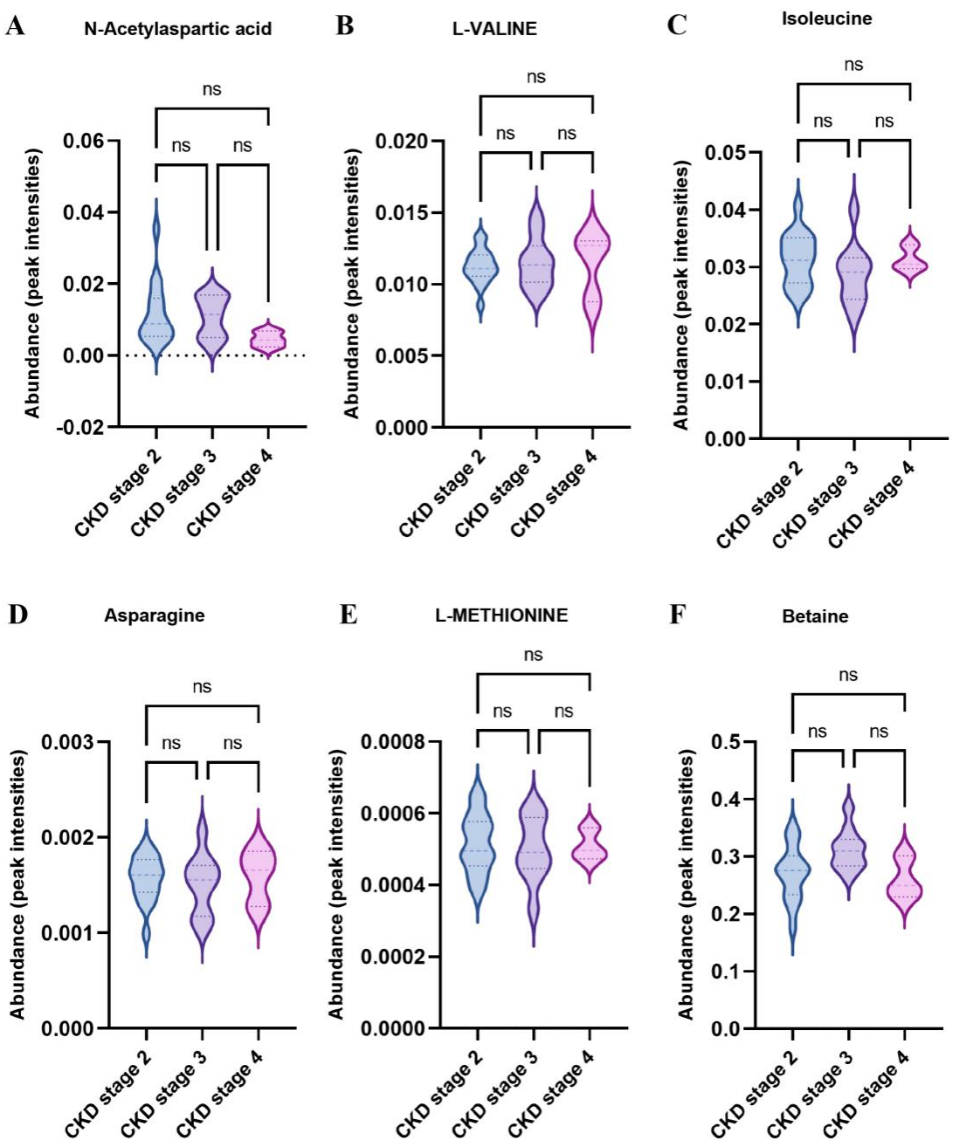

Figure S6. Detection of differentially expressed metabolites based on different CKD stages.

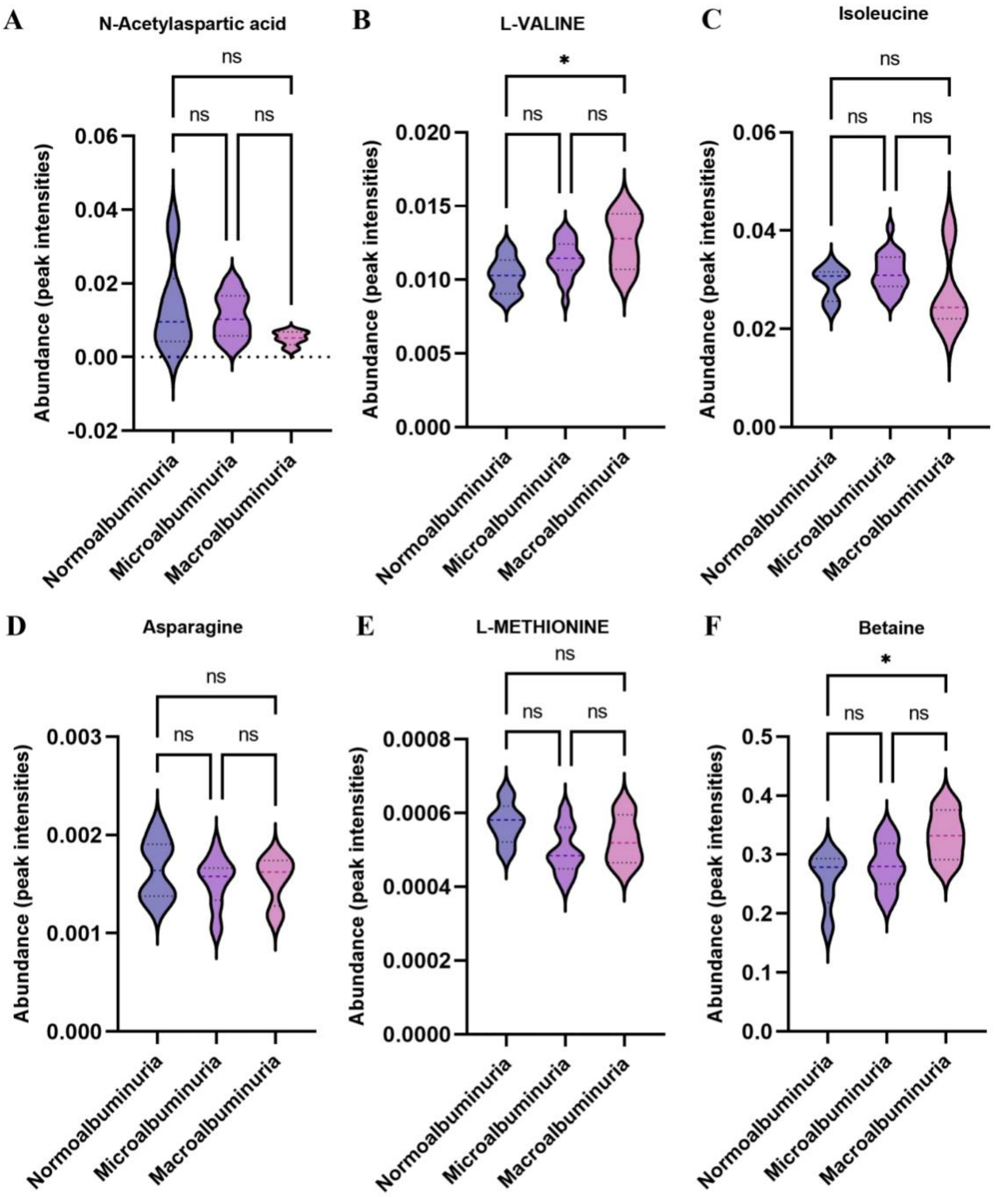

Figure S7. Detection of differentially expressed metabolites based on different degree of proteinuria.

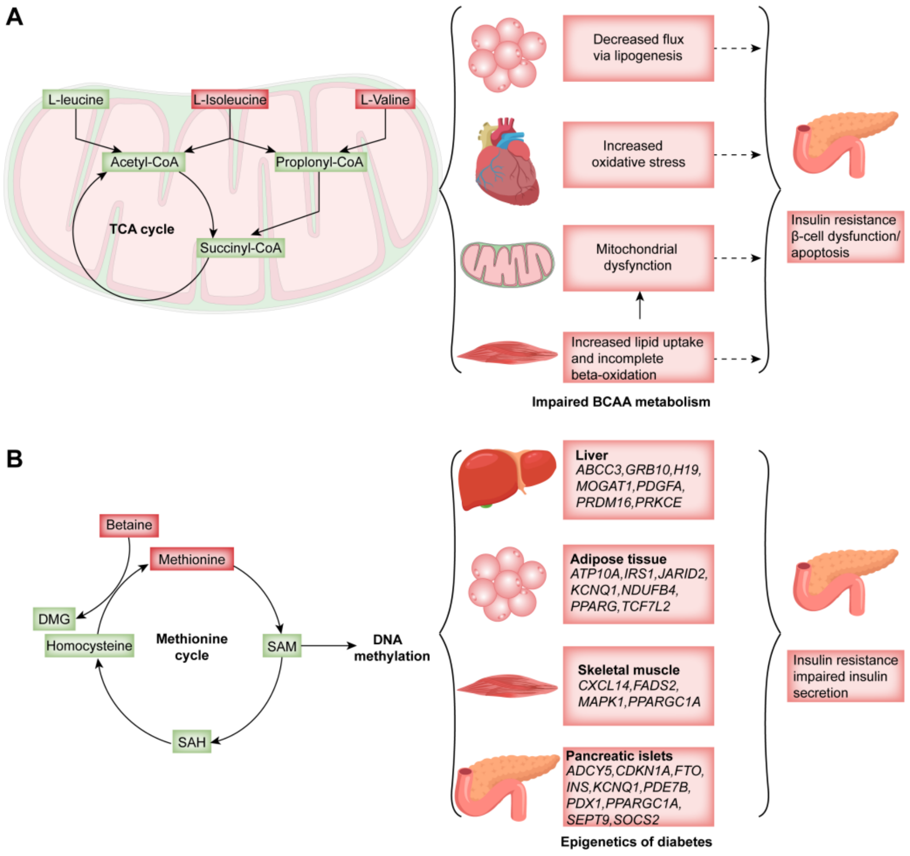

Figure S8. The disturbance of amino acid metabolism pathway in the progression of diabetes. (A) L-valine and L-isoleucine in BCAA catabolism pathway were gradually up-regulated, which were associated with insulin resistance through a variety of potential mechanisms. (B) Methionine and betaine in the methionine cycle and DNA methylation pathways were gradually up-regulated, and increased DNA methylation and decreased expression of these genes were associated with impaired insulin secretion.

Supplementary Table S1. The metabolites in Health, T2DM, and DKD subjects

Metabolites	Change up	Health vs T2DM			Health vs DKD			T2DM vs DKD		
		Fold change	VIP	P value	Fold change	VIP	P value	Fold change	VIP	P value
N-Acetylaspartic acid	down	0.54	1.33	<0.001	0.28	1.80	<0.001	0.52	1.08	<0.001
Coumaroyl + C6H9O8	up	5.97	1.53	<0.001	65161.15	2.42	<0.001	10922.87	2.29	<0.001
NCGC00385656-01	up	3.23	1.63	<0.001	598.24	2.38	<0.001	185.00	2.42	<0.001
L-VALINE	down	0.73	1.44	<0.001	0.83	1.23	<0.001	0.61	1.58	<0.001
Betaine	down	0.76	1.23	<0.001	0.79	1.19	<0.001	0.60	1.50	<0.001
Asparagine	down	0.69	1.41	<0.001	0.80	1.22	<0.001	0.55	1.55	<0.001
L-METHIONINE	down	0.71	1.20	<0.001	0.84	1.05	<0.001	0.59	1.55	<0.001
Isoleucine	down	0.70	1.41	<0.001	0.69	1.69	<0.001	0.48	1.69	<0.001
7-Diethylamino-4-methylcoumarin	up	19.52	1.21	<0.001	850.88	1.46	<0.001	16607.81	1.75	<0.001
Matairesinol	up	1.71	1.28	<0.001	33.16	1.88	<0.001	56.76	1.52	<0.001
NCGC00385072-01	up	1.75	1.19	<0.001	68.14	2.00	<0.001	119.09	1.61	<0.001
N-Methyl-2-pyrrolidone	up	2.24	1.66	<0.001	38.85	2.14	<0.001	86.91	1.74	<0.001
Trinexapac-ethyl	up	1.77	1.26	<0.001	61.26	2.09	<0.001	108.70	1.68	<0.001
4-(Dimethylamino)pyridine	down	0.71	1.11	<0.001	0.76	1.22	<0.001	0.54	1.47	<0.001
Nicotinic acid mono nucleotide	down	0.72	1.65	<0.001	0.85	1.01	<0.001	0.61	1.49	<0.001
N-Methylaniline	down	0.13	1.09	<0.001	0.48	1.68	<0.001	0.06	1.11	<0.001
Pesticide2_Oxamyl	down	0.72	1.45	<0.001	0.84	1.08	<0.001	0.61	1.52	<0.001
Thiobispropanoic acid	down	0.72	1.64	<0.001	0.85	1.03	<0.001	0.62	1.49	<0.001
